# Going Virtual When The Doors Close: Addressing Geriatric Workforce Training Needs During A Pandemic

**DOI:** 10.1093/geroni/igab046.405

**Published:** 2021-12-17

**Authors:** Linda Edelman, Gail Towsley, Timothy Farrell

## Abstract

The focus of our Geriatric Workforce Enhancement Program (GWEP) is to enhance long-term services and support (LTSS) and primary care healthcare workforce capacity through interprofessional education (IPE) and to increase patient, family, and caregiver engagement. When it became evident that LTSS settings, schools, and communities were going to be adversely impacted by the COVID-19 pandemic for the unforeseeable future, our GWEP quickly pivoted to address new challenges and initiate technology to continue our programs. In this symposium, we describe four programs implemented or revised during the COVID-19 pandemic. We utilized CARES (Coronavirus Aid, Relief and Economic Security) funding to develop a 3-part Project ECHO on utilizing telehealth in LTSS settings. We pivoted quarterly Fireside Chats – community-based educational programs held at partnering LTSS settings for older adults and caregivers – to bi-weekly and now monthly webinars addressing topics relevant to COVID-19 and combatting social isolation. Because students could no longer attend an in-person IPE course introducing them to long-term care, we revised the course to be online with a partnering nursing home participating in an interactive mock care conference. Finally, a 2-semester undergraduate Honors College project-based course introducing students to successful aging utilized virtual activities to expose students to the challenges of hospice care during a pandemic. With these adaptations, as well as activities that advocated for, and supported, LTSS settings and older adults, our GWEP program was able to continue to provide education and support to the setting and individuals most impacted by COVID-19.

